# Near Infrared Fluorescence (NIRF) Molecular Imaging of Oxidized LDL with an Autoantibody in Experimental Atherosclerosis

**DOI:** 10.1038/srep21785

**Published:** 2016-02-25

**Authors:** Ramzi Y Khamis, Kevin J. Woollard, Gareth D. Hyde, Joseph J Boyle, Colin Bicknell, Shang-Hung Chang, Talat H Malik, Tetsuya Hara, Adam Mauskapf, David W Granger, Jason L. Johnson, Vasilis Ntziachristos, Paul M Matthews, Farouc A Jaffer, Dorian O Haskard

**Affiliations:** 1 Vascular Sciences Section, National Heart and Lung Institute, Imperial College London, London, United Kingdom; 2 Department of Medicine, Imperial College London, London, United Kingdom; 3 Department of Surgery & Cancer, Imperial College London, London, United Kingdom; 4 Cardiovascular Research Center and Cardiology Division, Massachusetts General Hospital, Harvard Medical School, USA; 5 Biopharm R&D, GlaxoSmithKline, Stevenage, United Kingdom; 6 School of Clinical Sciences, University of Bristol, United Kingdom; 7 Brain Sciences, Department of Medicine, Imperial College London, London, United Kingdom

## Abstract

We aimed to develop a quantitative antibody-based near infrared fluorescence (NIRF) approach for the imaging of oxidized LDL in atherosclerosis. LO1, a well- characterized monoclonal autoantibody that reacts with malondialdehyde-conjugated LDL, was labeled with a NIRF dye to yield LO1-750. LO1-750 specifically identified necrotic core in *ex vivo* human coronary lesions. Injection of LO1-750 into high fat (HF) fed atherosclerotic *Ldlr*^−/−^ mice led to specific focal localization within the aortic arch and its branches, as detected by fluorescence molecular tomography (FMT) combined with micro-computed tomography (CT). *Ex vivo* confocal microscopy confirmed LO1-750 subendothelial localization of LO1-750 at sites of atherosclerosis, in the vicinity of macrophages. When compared with a NIRF reporter of MMP activity (MMPSense-645-FAST), both probes produced statistically significant increases in NIRF signal in the *Ldlr*^−/−^ model in relation to duration of HF diet. Upon withdrawing the HF diet, the reduction in oxLDL accumulation, as demonstrated with LO1-750, was less marked than the effect seen on MMP activity. In the rabbit, *in vivo* injected LO1-750 localization was successfully imaged *ex vivo* in aortic lesions with a customised intra-arterial NIRF detection catheter. A partially humanized chimeric LO1-Fab-Cys localized similarly to the parent antibody in murine atheroma showing promise for future translation.

Atherosclerosis is a chronic inflammatory disease of arteries that involves both the innate and adaptive immune systems^1^. It remains clinically challenging to determine which atherosclerotic plaques are prone to rupture and result in acute clinical manifestations, such as myocardial infarction or stroke^2^. Intravascular imaging techniques such intravascular ultrasound (IVUS) with virtual histology analysis (IVUS-VH), optical coherence tomography (OCT), and newer non-invasive techniques such as coronary artery CT have made considerable progress in defining atherosclerotic plaque morphology^3^,^4^. Furthermore, the ability of morphological analysis to predict plaque progression can be augmented by identifying local regions of low endothelial shear stress^5^. However, even in combination, the ability of these techniques to identify plaques that will rupture remains limited^5^. In this context, near infrared fluorescence (NIRF) optical molecular imaging offers a new approach to the evaluation of coronary plaque biology, especially in light of the development of intravascular NIRF detectors^6^.

Fluorescence molecular tomography (FMT) is a powerful NIRF preclinical translational imaging modality that allows simultaneous quantitative molecular imaging in small animals of one or more tracers labeled with dyes operating in distinct near infrared (NIR) spectra^7^. When combined with CT, FMT has revolutionised the ease with which molecular targets may be studied non-invasively, offering a non-radioactive near equivalent to single-photon emission CT (SPECT) and positron emission tomography (PET)^8^,^9^.

Oxidized LDL (oxLDL) is a prime target for the molecular imaging of atherosclerosis, not only because it is instrumental in plaque pathobiology but also because its presence may reflect plaque vulnerability^10^,^11^,^12^,^13^. Imaging of oxLDL in atherosclerosis has been reported in preclinical *ex vivo* studies using anti-oxLDL antibodies conjugated to single-photon emitting isotopes^14^,^15^,^16^, and more recently *in vivo* with anti-oxLDL-labeled MR-contrast agents^17^,^18^,^19^. Apart from a non-quantitative pilot study using a polyclonal antibody^20^, optical molecular imaging of oxLDL in atherosclerosis, alone or in conjunction with other molecular probes, has not yet been reported.

LO1 is a spontaneously arising IgG3k germline encoded natural monoclonal autoantibody isolated in our laboratory. It was selected for reacting with copper-oxidized LDL *in vitro,* and subsequently found to recognize LDL conjugated with malondialdehyde (MDA-LDL)^21^. LO1 recognizes antigen in tissue sections of mouse and human atherosclerosis upon immunocytochemical staining, with the binding to sections prevented by free MDA-LDL^21^.

We have now generated a near infrared fluorescent derivative of LO1 to enable FMT NIRF imaging of oxLDL in murine atherosclerosis, and have compared its uptake in the same animals with that of a matrix metalloproteinase (MMP) -activatable fluorescence probe. We then extended the study to show the potential of LO1 for intra-arterial NIRF molecular imaging of oxLDL in the rabbit. In a further translational step, we have developed a human-mouse chimeric Fab version of LO1 and validated its targeting profile in the mouse.

## Results

### LO1 binds antigen in human, mouse and rabbit atherosclerosis *ex vivo*

**Figure 1:**
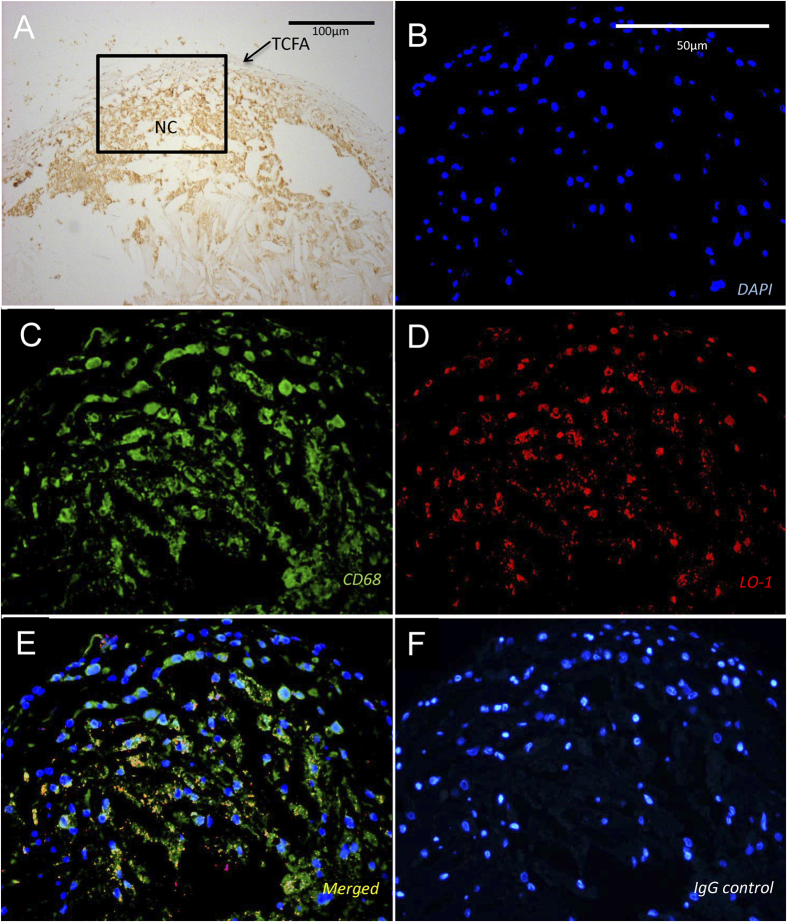
LO1 binds to determinants in the necrotic core in human coronary atherosclerotic lesions. (**A**) Advanced human coronary plaque stained for macrophages with anti-CD68 (brown). The lesion displays the typical characteristics of a vulnerable plaque with a large necrotic core (NC), a thin cap fibro atheroma (TCFA), and a large accumulation of CD68 positive macrophages; (**B–D**) show serial sections stained with (**B**) TOPRO nuclear stain (blue), (**C**) anti-CD68 (green) and (**D**) LO1 (red). LO1 clearly stains non-cellular aggregates in the necrotic core as well as co-localizing to macrophages in a merged image (**E**). (**F**) Shows negative signal obtained with IgG3 control antibody staining (red) merged with nuclear staining with TOPRO (blue).

To confirm the translational relevance of developing NIRF imaging with LO1 in experimental atherosclerosis, we confirmed with indirect immunohistochemistry (IHC) that LO1 reacts with antigen in human coronary atherosclerosis. Figure 1 shows LO1 staining diffusely in a CD68 positive macrophage-rich lesion with a thin fibrous cap and a large necrotic core. The staining pattern also suggests the identification of free aggregates, with some co-localization to macrophages. Staining with control IgG3 was negative. Overall, the localization of the unlabeled antibody to coronary lesions was in a pattern similar to that we have previously shown in human carotid atherosclerosis^21^.

We also found by IHC that LO1 binds antigen in atherosclerotic lesions in high fat-fed rabbits, staining particularly the macrophage-rich areas identified by RAM-11 staining. LO1 also stained free deposits within the plaque. There was no clear localization of LO1 to vascular smooth muscle cells (VSMCs) as identified by anti-smooth muscle actin (αSM-actin) (Supplementary Figure 1A–F). Notably, LO1 stained the shallow macrophage-rich plaque in thin cap fibro- atheroma lesional areas (Supplementary Figure 1G,H) and deeper lesional areas in moderate lesions sparing the thick fibro-atheroma area (Supplementary Figure 1I,J)

**Figure 2:**
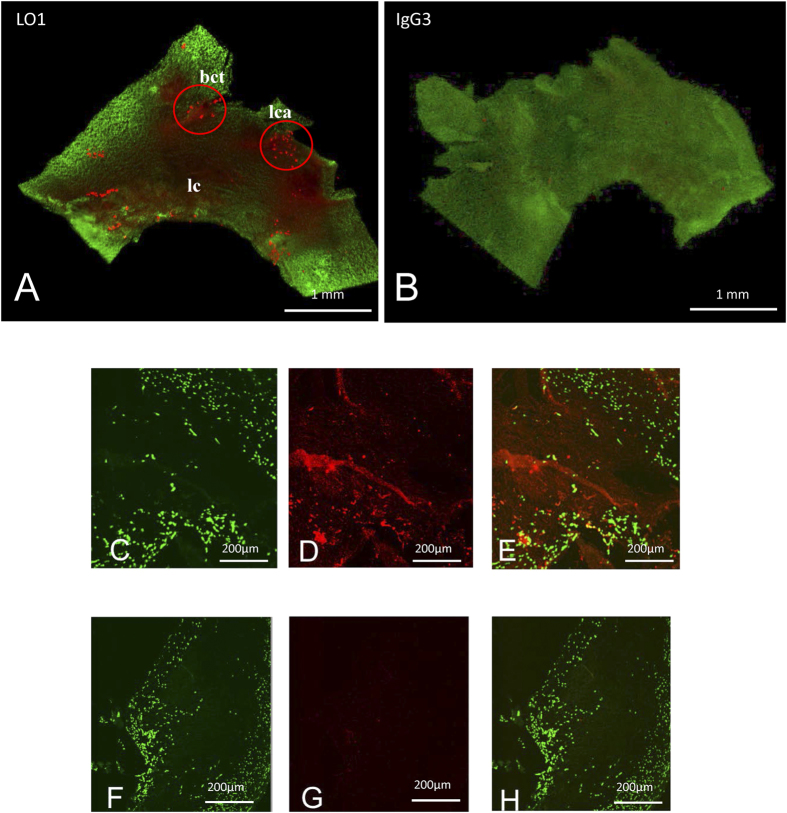
Staining of mouse arterial tissue with directly labeled LO1-750. (**A,B**) Direct staining of *en face* preparations of aortic arches from *Ldlr*^−/−^ mice, using (**A**) LO1-750, or (**B**) isotype control IgG3-750. Staining with the VivoTag-S 750-MAL labeled antibodies is red and nuclei counterstained with SYTO-24 are green. LO1-750 staining was diffuse and concentrated in aggregates at bifurcations of large branches (circles: bct = brachiocephalic trunk, lca = left carotid artery) and on the lesser curvature (lc) (**C–H**) show representative consecutive serial sections next to the necrotic core of a human carotid atherosclerotic plaque. (**D**) shows diffuse and focal staining with LO1-750 (red), and (**G**) shows negative staining with control IgG-750; (**C,F**) show nuclear counterstaining with SYTO-24 (green); (**E,H**) are merged images.

We next labeled the LO1 antibody with a NIRF dye, demonstrated retention of antigen binding (data not shown) and used confocal microscopy to demonstrate binding to lesions *in situ*. When applied to mildly permeabilized *en face* preparations of the aortic arch from an *Ldlr*^−/−^ mouse, LO1 labeled with VivoTag-S 750-MAL (designated LO1-750) stained the aortic arch diffusely, as well as concentrating in aggregates on the lesser curve and at large artery branch points (Fig. 2A). No staining was seen with equivalently-labeled isotype-matched IgG3κ (designated IgG3-750), demonstrating specificity of LO1-750 binding (Fig. 2B). LO1-750 staining of human carotid atherosclerotic plaque showed similar specific diffuse and focal staining (Fig. 2C-H) to that previously shown with the unlabeled antibody^21^. As was the case in our previous study with unconjugated LO1^21^, binding of LO1-750 to atherosclerotic lesions was inhibited by MDA-LDL in solution (data not shown).

### Clearance of LO1-750 after injection in mice

**Figure 3:**
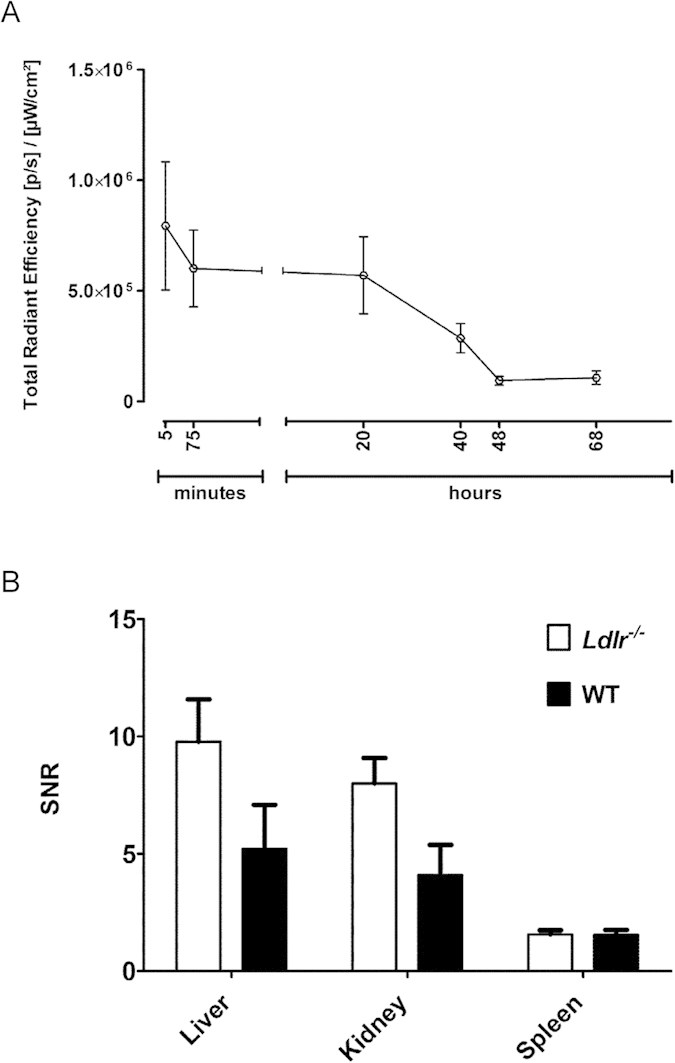
Blood clearance and organ distribution of LO1-750. (**A**) Shows the clearance from the blood of LO1-750 as determined by serial bleeds of *Lldr*^−/−^ and WT mice (n = 3/group). There was no difference in clearance between the two groups, and data points are mean + SEM from the mice combined. The clearance displayed a two-phase decay pattern with a fast and a slow phase. The overall half-life (t_1/2_) of LO1-750 in the circulation was estimated at 21 hours; (**B**) shows equivalent *ex vivo* organ signal-to-noise ratio (SNR) of LO1-750 in *Ldlr*^−/−^ and WT mice (n = 4/group) with most of the antibody localizing in liver, kidneys and spleen in both groups. Data points are mean + SEM.

We next assessed the blood clearance and organ distribution of LO1-750 in mice following intravenous (*iv*) injection. Heparinized blood samples were taken by serial tail bleeds and were imaged *ex vivo* for epifluorescence using IVIS Spectrum. The blood clearance of LO1-750 demonstrated a two-phase decay pattern, with no significant difference between *Ldlr*^−/−^ and wild-type (WT) animals over 68 hours. There was a rapid immediate phase of clearance lasting minutes, followed by a slower phase with detectable levels of LO1-750 remaining in the circulation over the duration of the experiment, as expected for a 150kD antibody (Fig. 3A). The overall half-life of *iv*-injected LO1-750 was estimated as 21 hours. The organ distribution of LO1-750 at 4 hours after injection was broadly equivalent in *Ldlr*^−/−^ and WT animals, with localization particularly in liver, kidneys, and spleen (Fig. 3B). The organ distribution of IgG3-750 control was similar to that of LO1-750 (data not shown). Based on the blood clearance kinetics, we performed pilot non-quantitative, live animal imaging with IVIS Spectrum/CT at 4 hours after *iv* injection. This showed that LO1-750 (Supplementary Figure 2A) but not IgG3-750 (Supplementary Figure 2B) localized to a region of interest (ROI) encompassing the ascending thoracic aorta and aortic arch, including some areas of calcified atherosclerosis as seen on a separate contrast-enhanced CT (Supplementary Figure 2C,D). We also performed selected *ex vivo* NIRF imaging on extracted aortae to demonstrate that the fat surrounding the extracted aortae does not emit a fluorescent signal in LO1-750 injected animals (Supplementary Figure 2E).

### Quantitative analysis of LO1-750 imaging of atherosclerosis in mice

**Figure 4:**
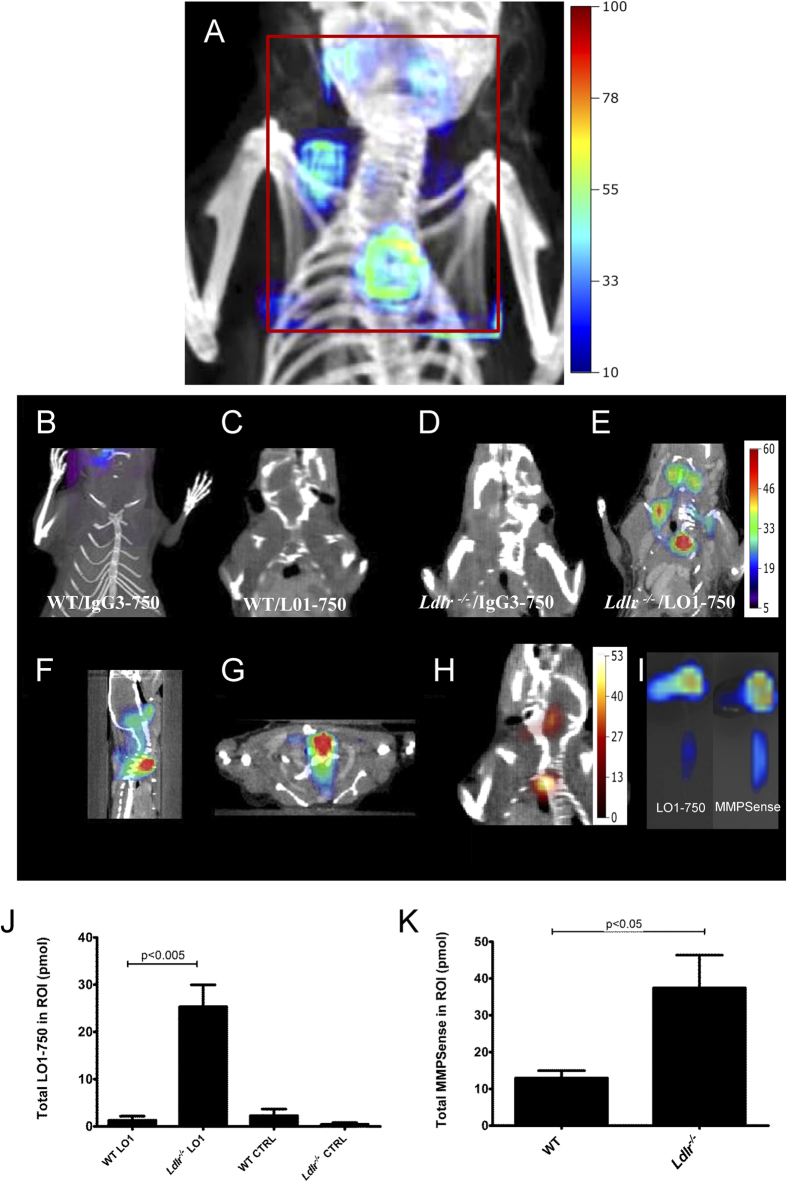
FMT/CT imaging and quantification of LO1-750 localization in *Ldlr*^−/−^ mice. (**A**) An *Ldlr*^−/−^ mouse was injected with 50 μg LO1-750 and imaged after 4 hours. The figure shows an FMT/CT reconstruction of the localization of LO1-750 in the ROI encompassing the aortic proximal aorta and it branches, with signal arising from the general region that includes aortic arch as well as brachiocephalic trunk/ right subclavian and both upper carotids. The box illustrates the position of the 3D ROI used for subsequent quantification. The color scale bar represents quantifiable signal intensity in nM of LO1-750. Four groups of animals (n = 6/group) are represented in coronal views in (**B**–**E**): (**B,C**) WT controls and (**D,E**) age-matched *Ldlr*^−/−^ mice fed a HF diet for 42 weeks. (**B,D**) were injected with control IgG3-750, and (**C,E**) were injected with the equivalently-labeled LO1-750 (both 50 μg/injection). All animals also received MMPSense FAST (44 nmol/kg). Mice were imaged after 4 hours Only the *Ldlr*^−/−^ mice injected with LO1-750 displayed a clear signal in the ROI (**E**). The color scale bar is in nM from blue (low signal intensity) to red (high signal intensity); (**F,G**) show the images in (D) in sagittal and axial views respectively; (**H**) demonstrates the MMPSense image in a representative *Ldlr*^*−/−*^mouse. The color scale bar is nM from black (low signal intensity) to orange (high signal intensity); (**I**) shows positive LO1-750 and MMPSense signals in the aortic arch and thoracic aorta on FMT epifluorescence imaging *ex vivo* (same scale as (**A–F**); (**J**) provides the quantification of LO1-750 signal in *Ldlr*^−/−^ mice compared to the other groups, and (**K**) shows the higher MMPSense signal in *Ldlr*^−/−^ *vs*. WT controls. Values are mean 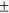 SEM.

We proceeded to imaging LO1-750 localization using FMT/CT, a modality that allows for three dimensional (3D) acquisition and reconstruction of NIRF signal and provides absolute quantification within in a defined 3D ROI^7^,^22^. We compared groups of *Ldlr*^−/−^ mice and WT age-matched controls, with each mouse receiving *iv* injections of either LO1-750 or IgG3-750 (both 1.5 mg/kg). For comparison, all mice also received MMPSense-645-FAST (henceforth designated MMPSense), an agent that is optically silent until cleaved by disease-related MMPs, including MMP 2, 3, 7, 9, 12, and 13. Figure 4A shows in a coronal plane of an *Ldlr*^−/−^ mouse injected with LO1-750 the position of the ROI used for quantifying molecular probe localization. FMT allowed the reconstructions to be viewed as 360° rotating 3D images (Supplementary Movie 1) or in coronal (4E), sagittal (4F) and axial (4G) 2 D planes (Supplementary Movie 2). Besides obvious uptake at the ROI encompassing the base of the aorta and the aortic arch, focal imaging was also evident in the regions of the carotid and subclavian arteries. As shown in representative images in Fig. 4B–E, and quantitatively as a histogram in Fig. 4J, there was a significantly higher level of LO1-750 uptake in *Ldlr*^−/−^ mice compared to WT animals (25.3 ± 4.6 pmol *vs.* 1.3 ± 0.9; mean ± SEM; n = 6/group; p = < 0.005). Moreover, IgG3-750 accumulated at a substantially lower level than LO1-750 in ROIs of *Ldlr*^−/−^ mice (0.4 ± 0.3 pmol; p < 0.05 for each of the IgG3-750 groups in comparison to LO1-750 in the *Ldlr*^−/−^ group).

MMPSense activation occurred in the same predefined ROI of *Ldlr*^−/−^ mice (Fig. 4H), and was the same in animals receiving LO1-750 or IgG3-750 (p = 0.32, Mann-Whitney test), providing a means to non-invasively validate the equivalent pathology in the two groups. The level of MMP activation measured in the ROIs was 37.4 ± 8.9 pmol in the *Ldlr*^−/−^ *vs.* 13 ± 2.0 pmol in WT mice (p < 0.05) (Fig. 4K). Comparing L01-750 with MMPSense, LO1-750 therefore gave a superior *Ldlr*^−/−^ to WT NIRF signal ratio in the ROI (19.3 vs 2.8, p = 0.03). We also used the FMT imaging system to perform selective fluorescence reflective imaging (FRI) on aortae extracted *post mortem*. Figure 4I demonstrates both the *ex vivo* LO1 and MMPSense signals in the aortic root and thoracic aorta from an *Ldlr*^−/−^ mouse.

### Validation of LO1-750 targeting in mice using confocal microscopy

**Figure 5:**
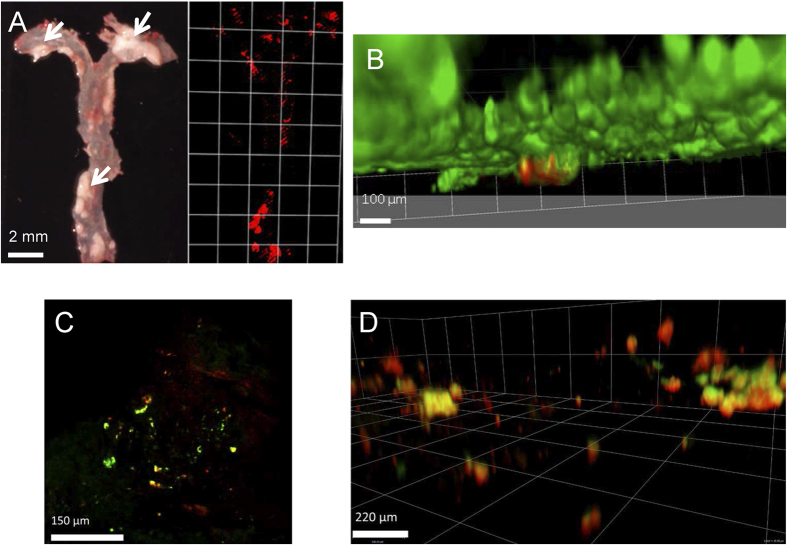
*En face* confocal microscopy of the aorta of an *Ldlr*^−/−^ mouse following *iv* injection of LO1-750. An *Ldlr*^−/−^ mouse received LO1-750 (50 μg) by *iv* injection, 4 hr after which the aorta was removed for *ex vivo* confocal microscopy analysis. (**A**) white light image displaying atherosclerotic lesions (arrows) in the aortic arch and its bifurcations, as well as the abdominal aorta, with corresponding *en face* confocal microscopy showing LO1-750 accumulation (red); (**B**) 3D imaging revealing an accumulation of LO1-750 (red) underneath endothelium identified by phycoerythrin-labeled anti-CD31 antibody injected *iv* ten min before humanely killing; (**C)** LO1-750 (red) staining in relation to MOMA*-*stained macrophages (green = macrophages; yellow = merged); (**D**) 3D imaging of *en face* section of aorta studied in (**C**) demonstrating LO1-750 (red) in relation to macrophages (green = macrophage; yellow = merge). Note the distinct aggregates of material bound by LO1-750.

Confocal microscopy was performed on aortae extracted from animals that had received *iv* LO1-750. Figure 5A demonstrates the *en face* appearance of an *Ldlr*^−/−^ aorta, with lesions in the inner curvature as well as the abdominal aorta. The adjacent panel to the right shows LO1-750 accumulation (red) as imaged by Z and tile stacking in the 750–800 nm emission channel, co-localizing with atherosclerotic lesions. By injecting simultaneously a phycoerythrin (PE)-conjugated anti-CD31 antibody to identify vascular endothelium, we demonstrated that LO1-750 localized within the arterial wall beneath endothelium (Fig. 5B and Supplementary Movie 3). High magnification studies showed LO1-750 localization in the vicinity of MOMA-2-positive macrophages (Fig. 5C,D). Taken together, these observations established that LO1-750 penetrates the endothelium and localizes in macrophage-rich areas of the arterial wall.

### LO1-750 FMT imaging quantifies lesion progression

**Figure 6:**
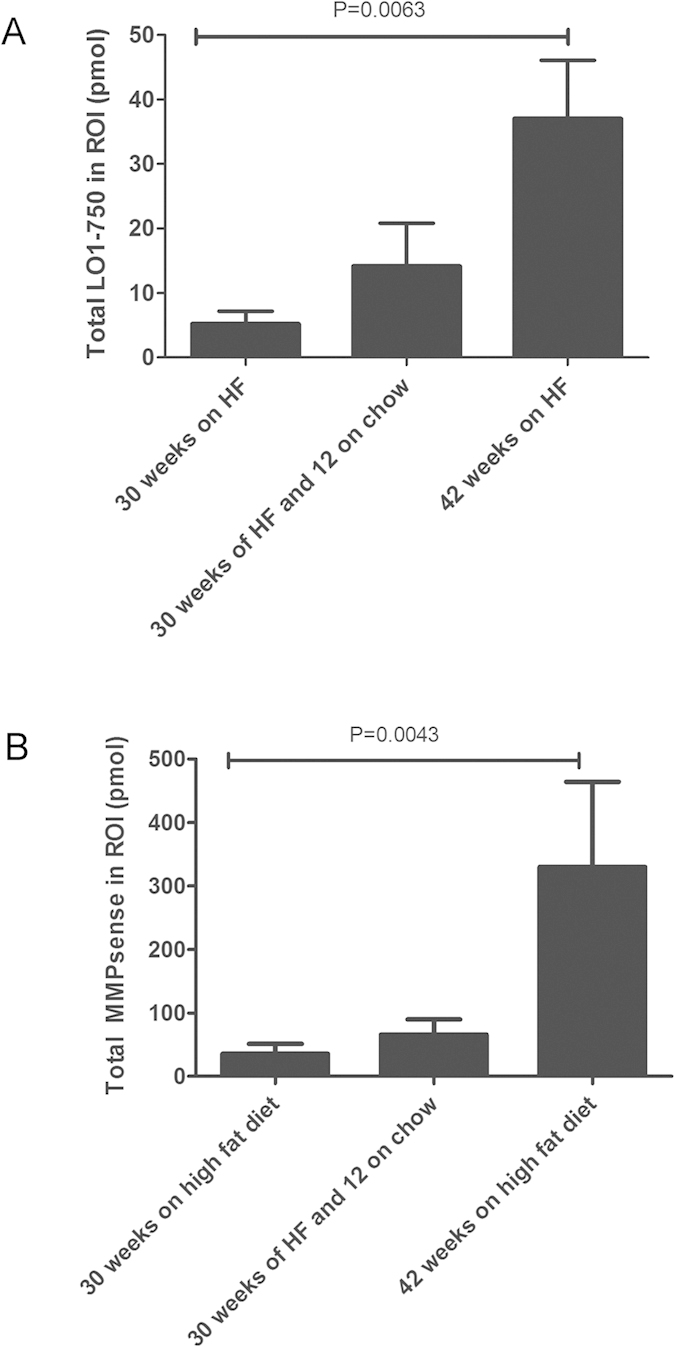
Quantification using FMT of LO1-750 and MMPSense targeting in mice in relation to duration of diet. Group I were fed a high fat diet for 30 weeks; Group II were fed a HF diet for 30 weeks followed by 12 weeks on chow; and Group III were fed a high fat diet for 42 weeks. Each mouse received 50 μg LO1-750 and 2.4 nmol MMPSense by *iv* injection and was imaged after 4 hours (**A**) demonstrates significantly more LO1-750 uptake in Group III vs. Group I, with a clear linear trend between the three groups (R square 0.42; p = 0.004); (**B**) shows that MMPSense gave a similar difference between Groups I and III but a weaker linear trend overall (R square 0.29; p = 0.02). Each group was composed of six mice.

We next performed a second FMT study with *Ldlr*^−/−^ mice to determine whether LO1-750 imaging could distinguish different levels of disease. Mice were given a HF diet for 30 weeks and were then divided into three groups (n = 6 in each group). Group I (baseline) was euthanized, whereas Group II (halted progression) was switched to low fat (LF) laboratory chow for a further 12 weeks. A third group was maintained on the HF diet for a further 12 weeks (Group III, advanced progression). Mice were then imaged with both LO1-750 and MMPSense as above. Figure 6A demonstrates that Group III had significantly more LO1-750 uptake at the ROI *vs.* Group I (37.04 ± 9.030 pmol *vs* 5.228 ± 1.9 pmol, n = 6/group; p < 0.05). Furthermore, when testing for linear trend between the three groups, LO1-750 demonstrated an R squared of 0.42 (p = 0.004). MMPSense also detected a significant difference between groups I and III (35.86 ± 15.42 pmol *vs.* 329.7 ± 134.6 pmol, p < 0.05) (Fig. 6B). There was, however, a weaker R squared when testing for linear trend between the three groups of 0.29 (p = 0.02), suggesting that MMPSense and LO1-750 indicate different and possibly complementary lesional characteristics.

### NIRF intravascular imaging of oxLDL in rabbit atherosclerosis

**Figure 7:**
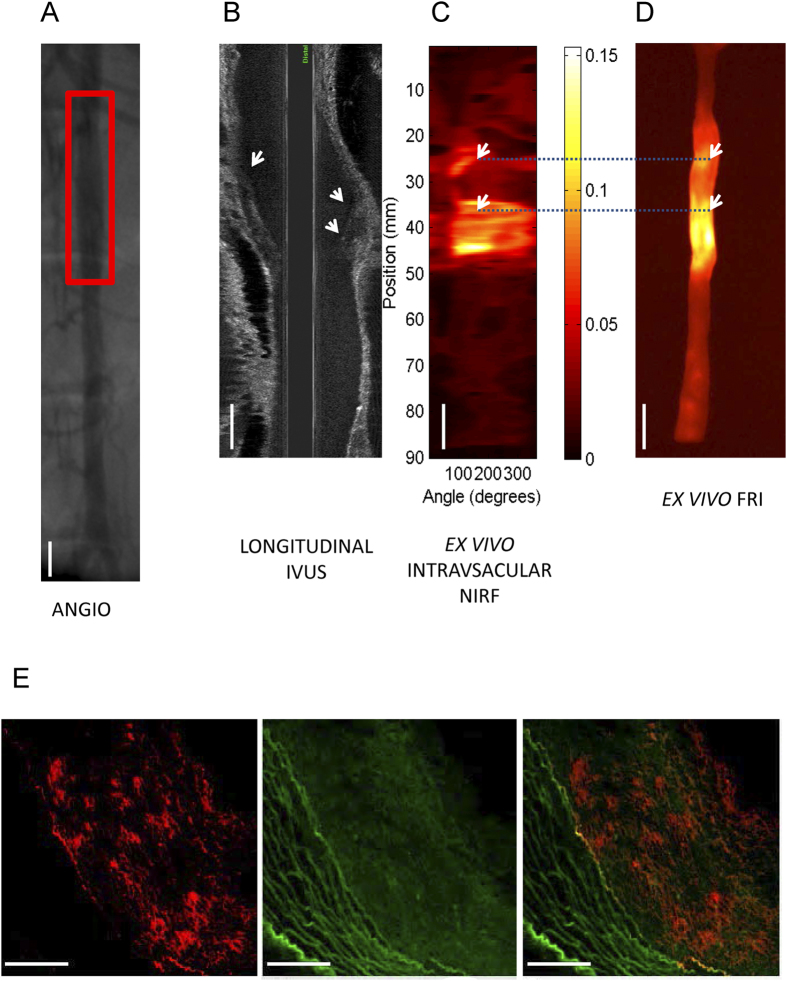
NIRF molecular imaging of oxLDL in atherosclerotic balloon injured rabbit abdominal aortae. LO1-750 (6.6 mg/kg in this example) was injected IV into rabbits with aortic atherosclerosis following balloon injury and HF feeding. (**A**) Shows conventional angiography of the rabbit aorta with the red box indicating the balloon injured area; (**B**) demonstrates the corresponding longitudinal IVUS catheter pullback through the aorta. The white arrows in (**B**) demarcate IVUS evidence of eccentric atherosclerotic plaque; (**C**) shows *ex vivo* two-dimensional (2D) intravascular NIRF imaging of *in vivo* LO1-750 localization after 21 hours. There are two distinct ‘hot spots’ seen on the intravascular 2D NIRF (white arrows in **(C,D**). The SNR of the 10 mm long plaque at the 34 mm to 35 mm mark was 86.4 and target to background ratio (TBR) was 4.8 (yellow/white: higher NIRF signal intensity, red/: lower NIRF signal intensity –arbitrary NIRF units); (**D**) demonstrates corresponding *ex vi*vo FRI (740/790 nm) with augmented NIRF LO1-750 activity in the two ‘hot spots’ seen on intravascular imaging. Corresponding lesions are linked by the dotted blue line; (**E**) demonstrates fluorescence microscopy of a cryosection from the larger lesion of the aorta shown in **(C–D**). The left panel in (**E**) is LO1-750 fluorescence (red) in the NIR channel showing focal and diffuse localization to constituents of plaque, the middle panel shows the distinctly different atherosclerosis autofluorescence pattern in the FITC channel (green), whilst the right panel is a merged image of both; Scale bars in represent 10 mm in (**A–D**) and 50 μm in (**E**).

NIRF molecular imaging of coronary atherosclerosis in human subjects is likely to require high resolution catheter-based approaches^20^. As LO1 binds to rabbit atherosclerotic lesions (Supplementary Figure 1), we next explored the ability of LO1-750 to localize to diseased rabbit aorta, a vessel of similar caliber to a human coronary artery and therefore potentially providing a model for catheter-based NIRF detection. Following *iv* injection of LO1-750, we imaged rabbit aortae with angiography, IVUS and 2D NIRF (n = 5). We focused on *ex vivo* intravascular 2D NIRF images of the resected aorta, as the background signal from residual circulating LO1-750 precluded accurate *in vivo* imaging. Figure 7A demonstrates the angiographic location of the balloon injury, with atherosclerotic plaque identified by IVUS in Fig. 7B. We found that the *ex vivo* NIRF signal obtained with the 2D intravascular catheter (Fig. 7C) was well matched to that obtained by FRI of the whole aorta (Fig. 7D). Validation of the deposition of LO1-750 in plaque was performed by *ex vivo* epifluorescence fluorescence microscopy (FM). Figure 7E shows a representative cryosection from the larger lesion of the aorta shown in 7C and 7D, with the distribution of LO1-750 in red and autofluorescence signal from the atherosclerotic lesion in green. This confirmed a similar type of deposition of LO1-750 to that seen in the mouse model, with punctate as well as more diffuse staining. Further *ex vivo* sections (Supplementary Figure 3) revealed partial localization to Oil Red O lipid staining (Supplementary Figure 3A,B), demonstrating that LO1 targets some but not all lipid forms. The high magnification images revealed a signal within the necrotic core and adjacent to the thin fibrous cap that is distinct from autofluorescence (Supplementary Figure 3C).

### Generation and characterization of LO1-Fab-Cys

**Figure 8:**
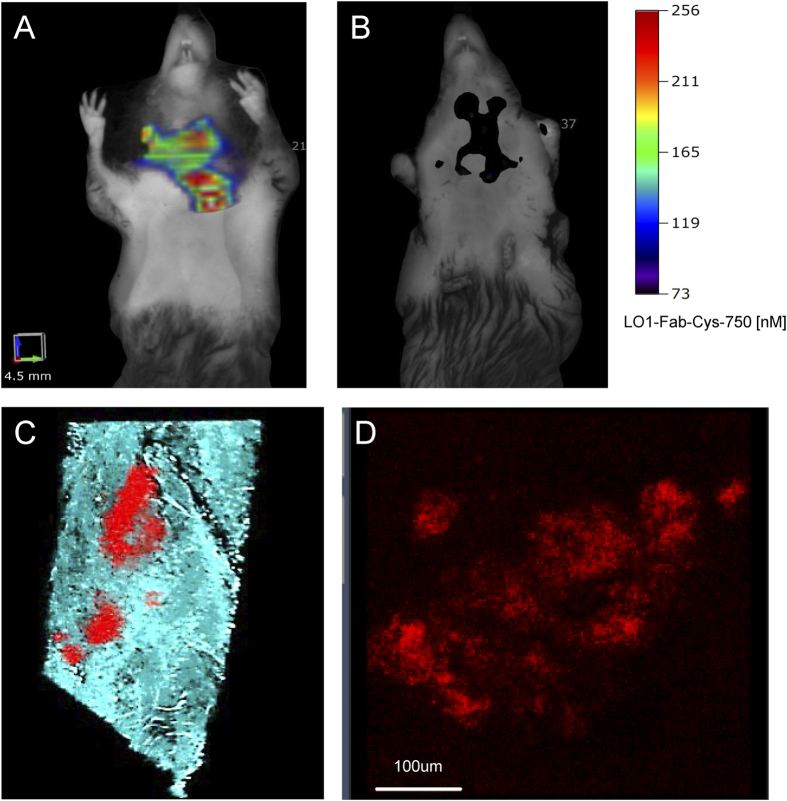
*In vivo* targeting with the human-mouse chimeric LO1-Fab-Cys-750. (**A–B**) FMT images 4 hr following *iv* injection of 50 μg LO1-Fab-Cys-750 (**A**) or 50 μg of inactivated LO1-Fab-Cys-750 control (**B**) into litter mate *Ldlr*^−/−^ mice, fed a HF diet for 42 weeks. LO1-Fab-Cys but not the inactivated control localized to the thoracic ROI; (**C**) the aorta from (**A**) was harvested and imaged *en face* by confocal microscopy. The image shows LO1-Fab-Cys-750 (red) in relation to subendothelial matrix (autofluorescence) (Em 520-590, blue) in the aortic arch. Higher magnification in (**D**) reveals similar pattern of LO1 localization as seen with whole antibody LO1-750 *en face* studies *ex vivo*.

As a step towards molecular engineering of LO1 constructs for clinical applications, we generated a recombinant 50 kD Fab construct (LO1-Fab-Cys) bearing LO1 VH and VL sequences fused respectively with human CH1 and CL domains, and with a free cysteine attached to the CH1 to facilitate ligand labeling (Supplementary Figure 4A). This chimeric Fab structure lacks the effector properties of intact antibodies provided by Fc, but retains the ability to react with oxLDL. Purity of the expressed LO1-Fab-Cys was established by gel electrophoresis (Supplementary Figure 4B), and mass spectrometry confirmed it to be 118 Da greater than the theoretical Fab mass (47627 Da vs 47509 Da) (Supplementary Figure 4C), consistent with the presence of the cysteine tag. Further characterization by ELISA demonstrated the relative reactivity of the Fab in comparison to LO1 when tested against anti-idiotype and laboratory-made MDA-LDL in a serial dilution series (Supplementary (Fig. 4D,E). We labeled LO1-Fab-Cys with VivoTag-S 750-MAL, and achieved a three-fold greater labeling capacity in comparison to the whole LO1 antibody per mg of protein. After confirming that the labeled LO1-Fab-Cys-750 construct retained function *in vitro*, we performed pilot *in vivo* FMT studies. These showed similar arterial localization of LO1-Fab-Cys-750 in the *Ldlr*^−/−^ mice as seen with LO1-750 (Fig. 8A), with no arterial localization in WT mice (not shown). No localization to ROI was detected using an inactivated LO1-Fab-Cys-750 (Fig. 8B). *Ex vivo* confocal microscopy demonstrated localization to atherosclerotic murine aorta and accumulation under the subluminal matrix identified by autofluorescence (Fig. 8C,D and Supplementary Movie 4). The appearance of the deposits of LO1-Fab-Cys was similar to that seen with LO1, with both punctate and diffuse lesional staining.

## Discussion

In this study we demonstrate for the first time the use of a monoclonal autoantibody combined with NIRF technology to image and quantify the accumulation of oxLDL in atherosclerosis *in vivo.* The LO1 antibody that we used reacts with LDL adducted with MDA, and was originally isolated from an *Ldlr*^−/−^ mouse that had not been subjected to active immunization, giving us the confidence that it is likely to target a biologically relevant endogenous entity. Furthermore, the genes encoding the LO1 heavy and light chain are essentially germline, indicating that it is a naturally occurring autoantibody^21^.

Importantly for translational purposes, we found that LO1 is broadly cross-reactive across species, with clear immunocytochemical evidence of antigen detection in mouse, rabbit and human atherosclerotic lesions. In each species, atherosclerosis tissue staining with LO1 indicated that it bound both diffusely-distributed and aggregated antigen, often in the vicinity of macrophages within the necrotic core. In the case of the mouse *en face* aortic preparations, aggregates stained by LO1 were distributed on the lesser curvature and also at arterial bifurcations in clusters, whilst the control antibody staining was completely negative. In rabbit histological studies, LO1 localized to macrophage-rich and not VSMC-rich areas.

The *in vivo* localization of LO1-750 in the mouse was validated with *ex vivo* fluorescence reflectance imaging, and *en face* confocal microscopy on whole aortae with Z stacking and tile scanning. These confirmed localization of the probe to atherosclerotic plaque within the diseased aortic arch and its bifurcations, as well as a weaker signal in plaque within the abdominal aorta. Furthermore, by co-injecting phycoerythrin-conjugated anti-CD31 to identify endothelial cells, we were able to establish that the injected antibody penetrated beneath endothelium. Three dimensional confocal imaging established that the *iv-*injected LO1-750 identified extracellular aggregates in the vicinity of macrophages. We believe that these aggregates may be particularly relevant pathophysiologically, as they are indicative that cellular and humoral debris clearance mechanisms have failed.

It was pertinent to demonstrate that the labeled LO1-750 could be imaged with more than one specific NIRF modality. Thus initially we selected non-quantitative IVIS Spectrum imaging as an imaging starting point and also used its epifluorescence capabilities to calculate the half-life and relative organ distribution of the labeled antibody. Once successful, we progressed to four-laser FMT imaging. The power of FMT lies in its ability to localize and construct 3D tomographic NIRF images with picomolar quantitative sensitivity, as well as simultaneously image up to four NIRF agents at different frequencies. It also readily allows for image fusion with anatomical micro-CT imaging using fiducial reference points^7^.

Using FMT/CT, we demonstrated that LO1-750 accumulates in the ROIs encompassing the aortic arch and its branches in *Ldlr*^−/−^ but not in WT mice, with the IgG3-750 isotype control giving very little signal in either. Furthermore, we tested the ability of LO1-750 to distinguish between different rates of atherosclerosis progression using three groups of *Ldlr*^−/−^ mice. A baseline group of atherogenic *Ldlr*^−/−^ mice fed HF for 30 weeks was easily distinguished from an advanced atherosclerosis group exposed to continued atherogenic stimulus by feeding them HF for a further 12 weeks. In contrast, a third group in which HF diet was stopped at 30 weeks and swapped to LF diet for a further 12 weeks gave an intermediate LO1-750 signal, indicative of intermediate oxLDL accumulation. This pattern of oxLDL accumulation was as expected, since *Ldlr*^−/−^ animals have raised cholesterol despite being on a LF diet.

We achieved complementary imaging of another component of atherosclerosis using an activatable probe of MMP activity. MMPSense was chosen as it had shown promise in other disease models^23^, and is known from *in vitro* work to be activated by MMPs in human plaque^24^. Furthermore, the use of MMPSense provided a convenient positive control for equivalency between groups that received LO1-750 *vs.* IgG3-750. Similar to LO1-750, MMPSense provided good discrimination between *Ldlr*^−/−^ and WT animals, and was also able to discriminate between early and late disease in the progression study. The key difference between MMPSense and LO1-750 was seen following withdrawal of high fat diet. In contrast to LO1-750, MMPSense displayed the strengths of an acute phase reporter and imaged the slower progression in MMP signal upon reduction of lipid intake. This ability of an intervention (withdrawal of HF diet) to separate molecular read-outs hints at the potential of multi-agent molecular NIRF imaging as an aid to plaque evaluation.

As a proof of concept that LO1 can enable intravascular NIRF imaging of oxLDL in coronary sized vessels, we undertook pilot studies using the 2D NIRF system coupled with IVUS, that has been used to image balloon injury and stent-induced inflammation in the HF fed rabbit model^25^,^26^. *Ex vivo* intravascular 2D NIRF imaging of the aorta immersed in saline demonstrated that intravenously injected LO1-750 could be detected at high resolution in IVUS-confirmed atheroma. This finding was substantiated by matching whole aorta fluorescence reflectance NIRF imaging *ex vivo* with the lesional areas identified by LO1 on intravascular NIRF. It should be noted that we also demonstrated that the LO1-750 deposition in atheroma was distinct in comparison to autofluorescence in the FITC channel. Furthermore, *ex vivo* fluorescence microscopy studies of freshly frozen sections showed a pattern of localization similar to that seen in LO1-750 injected mice, with lesion-specific punctate and diffuse staining within necrotic core.

There are obvious obstacles to using a native mouse antibody for clinical imaging, including immunogenicity, Fc-related inflammatory effector function, and a relatively long circulating half-life increasing background signal. Indeed, we found that residual labeled LO1-750 prevented reliable detection *in vivo* in the rabbit in the presence of blood up to 18 hours after LO1-750 injection. However, recombinant DNA technology now allows antibodies to be expressed in a number of formats to optimize tissue penetration, antigen avidity and background clearance^27^. We have successfully cloned and sequenced the LO1 heavy and light chain variable regions, allowing us to molecularly express the antibody. LO1-Fab-Cys was created as a cysteine-tagged human chimeric Fab construct and was shown its function to resemble that of LO1 *in vitro*. Moreover, our initial pilot data *in vivo* show that LO1-Fab-Cys targets atherosclerotic plaque in a similar manner to its parent antibody, establishing that LO1-750 localization was not Fc dependent.

Intravascular NIRF molecular targeting of oxidized LDL and other molecular targets in coronary artery disease would have a number of advantages over other modalities. These would include the lack of added radiation compared to using radionuclear reporters and much better spatial resolution compared to whole body approaches. Importantly, the ultimate goal for NIRF molecular targeting is to integrate specific multi-agent biological information with both high resolution morphological imaging as well as endothelial shear stress data obtained by existing catheter-based technologies (IVUS and OCT), thereby providing a much needed extra dimension for improving the prediction of individual plaque progression and instability^28^.

A limitation of our study is the difficulty in correlating the quantified signals obtained *in vivo* to the signals from extracted aortae. There is also always the possibility that some of the signal we have detected *in vivo* arises from extravascular tissue, although our *ex vivo* data suggest this is not the case.

Besides being a stepping stone towards clinical imaging, the development of molecular probes for atherosclerosis imaging will provide the opportunity for a new generation of pathophysiological studies in which the molecular composition of plaques can be explored longitudinally, either using FMT in rodent models or with intravascular detectors in larger animals (eg rabbits, pigs). Such studies will provide new insights into atherosclerosis biology, and may help in the evaluation of novel targets and therapeutics.

## Materials and Methods

An expanded Materials and Methods is shown the Supplementary section.

### Ethical approvals

The methods were carried out in accordance with the approved guidelines. All animal experimental protocols undertaken at Imperial College were ethically approved by Imperial College and were carried out under the authority of UK Home Office licenses. All human carotid endarterectomy specimens were collected with informed consent with Imperial College institutional and UK National Ethical approvals. Stored coronary sections were used from the Athero Express study as per the pre-specified ethical protocols with approvals from the participating hospital ethical committees: UMCU Utrecht and St. Antonius hospital Nieuwegein, Holland^29^. Rabbit studies were undertaken at Harvard University with Harvard institutional ethical approval and in accordance with US legislation. All animal studies comply with our collaborator GSK Policy “Care, Welfare and Treatment of Animals”.

### Antibodies

LO1 was purified from hybridoma supernatants by affinity chromatography on protein G sepharose and when needed using a specific anti-idiotype column. An ELISA was used to confirm functional reactivity of LO1 before and after labeling, using MDA-LDL or H3 (anti-LO1 idiotype) on the solid phase, as previously^21^. Antibodies were labeled with the NIRF fluorochrome VivoTag-S 750-MAL (PerkinElmer, Massachusetts USA) and retention of function confirmed by ELISA. Further details are in the supplementary section.

### Generation of the chimeric LO1-Fab-Cys construct

Constructs consisting of human CH1 and CL regions fused respectively to LO1 VH and VL DNA sequences were each cloned into a mammalian expression vector backbone in frame with a DNA sequence encoding an N-terminal secretory signal peptide. A cysteine tag was added to the heavy chain to facilitate conjugation. The vectors were transfected into HEK293/6E cells and maintained in suspension to allow expression of secreted LO1-Fab-Cys molecules. Detailed methodology is in the supplementary section.

### Mouse model of atherosclerosis

We used female LDL receptor deficient (*Ldlr*^−/−^) mice (Jackson Laboratories, Bar Harbor, Me) and C57BL/6 WT control animals, which were maintained in a specific pathogen-free environment. The *Ldlr*^*−/−*^ mice were transferred at 10 weeks of age on to a high fat diet, the composition of which has been published^30^. For all imaging studies, the WT mice were age-matched and fed a low fat laboratory chow diet.

For the progression study, three groups of *Ldlr*^*−/−*^ mice were placed on HF diet for 30 weeks and then either euthenized (Group I), switched to LF diet for 12 weeks (Group II), or maintained on HF diet for a further 12 weeks (Group III).

Mice were anaesthetized for all *in vivo* imaging studies with either integrated isoflurane anesthesia, or intraperitoneal anesthesia with ketamine + midazolam.

### Rabbit model of atherosclerosis

For initial immunohistochemistry (IHC) studies, we used female New Zealand White rabbits fed a diet containing 1% cholesterol from 6 weeks of age for 8 weeks to yield atherosclerotic plaques in the aortic arch that were rich in foamy macrophages. For the intravascular imaging model, we induced plaque rich in inflammatory cells in male New Zealand White rabbits by balloon injury, as previously described^31^, followed by a 1% high fat diet for one week and 0.3% high fat diet for 16 weeks.

### Human atherosclerosis sections

As well as fresh frozen sections from carotid endarterectomy samples, we used well-characterized lesions from human coronary sections as per the AtheroExpress study protocol^32^. Details are in the supplementary section.

### Confocal and epifluorescence microscopy

Tissues were stored at −80 °C before use and cryosectioned as described^33^. Mouse *en face* aortic preparations were fixed in 2% formalin and permeabilized with 0.5% triton. Confocal microscopy was performed with a Leica SP5 MP inverted confocal microscope. Localization of injected LO1 or control IgG3 in relation to endothelium was studied by intravenously injecting phycoerythrin (PE)-conjugated anti-CD31 to identify endothelium (Biolegend, San Diego, CA). Image analysis was undertaken using Volocity 3D Image Analysis Software (PerkinElmer, Massachusetts USA). *Ex vivo* epifluorescence imaging of rabbit aortae was performed with an Eclipse 90i microscope (Nikon Instruments, Melville, New York).

Further methods for studying mouse, human and rabbit sections are included in the supplementary methods section.

### Fluorescence imaging and CT in mice

Blood clearance, organ distribution and preliminary imaging studies used IVIS Spectrum (Caliper LifeSciences) together with micro-CT (Inveon PET-CT, Siemens). Subsequently we used an FMT 4000 system (PerkinElmer, Massachusetts USA) equipped with four lasers. Animals were injected with a VivoTag-S 750-MAL-conjugated antibody, as well as in selected experiments with MMPSense-645-FAST (PerkinElmer, MA) to evaluate MMP activity or AngioSense 680 (PerkinElmer, MA) to identify intravascular space. Contrast-enhanced high resolution CT localized the aortic root and major arteries in the neck and thorax to guide the placement of the region of interest (ROI) for the fluorescence activity map concomitantly obtained by FMT. Fluorescence and CT image fusion relied on fiducial markers present on the imaging cartridge, and used Amide 1.0.4 (Sourceforge.net).

### Fluorescence imaging in rabbits

Intravascular imaging was performed with a stand-alone ***two***-dimensional (2D) NIRF imaging device and catheter apparatus^25^,^34^. Detailed methodology is in the supplementary section.

### Statistical analyses

Statistical analyses were undertaken using Prism 5.0. (GraphPad, La Jolla, California). Differences between two groups were determined using the Mann-Whitney *U* test for unpaired observations, and the Wilcoxon matched-pairs signed-ranks test for paired observations. Among multiple groups, significance was assessed via the Kruskal-Wallis test. Variables are reported as mean ± SEM. A p value of < 0.05 was considered statistically significant. In the feeding study, post-test for linear trend was undertaken, and results expressed in R square value with a significant p value of < 0.05.

## Additional Information

**How to cite this article**: Khamis, R. Y. *et al.* Near Infrared Fluorescence (NIRF) Molecular Imaging of Oxidized LDL with an Autoantibody in Experimental Atherosclerosis. *Sci. Rep.* **6**, 21785; doi: 10.1038/srep21785 (2016).

## Supplementary Material

Supplementary Information

Supplementary Movie 1

Supplementary Movie 2

Supplementary Movie 3

Supplementary Movie 4

## References

[b1] Libby P., Ridker P. M. & Hansson G. K. Inflammation in atherosclerosis: from pathophysiology to practice. J Am Coll Cardiol 54, 2129–2138, doi: 10.1016/j.jacc.2009.09.009 (2009). 19942084 PMC2834169

[b2] Yla-Herttuala S. *et al.* Stabilisation of atherosclerotic plaques. Position paper of the European Society of Cardiology (ESC) Working Group on atherosclerosis and vascular biology. Thromb Haemost 106, 1–19, doi: 10.1160/th10-12-0784 (2011). 21670845

[b3] Tian J. *et al.* Distinct morphological features of ruptured culprit plaque for acute coronary events compared to those with silent rupture and thin-cap fibroatheroma: a combined optical coherence tomography and intravascular ultrasound study. J Am Coll Cardiol 63, 2209–2216, doi: 10.1016/j.jacc.2014.01.061 (2014). 24632266

[b4] Wykrzykowska J. J. *et al.* Longitudinal distribution of plaque burden and necrotic core-rich plaques in nonculprit lesions of patients presenting with acute coronary syndromes. JACC Cardiovasc Imaging 5, S10–18, doi: 10.1016/j.jcmg.2012.01.006 (2012). 22421223

[b5] Stone P. H. *et al.* Prediction of progression of coronary artery disease and clinical outcomes using vascular profiling of endothelial shear stress and arterial plaque characteristics: the PREDICTION Study. Circulation 126, 172–181, doi: 10.1161/circulationaha.112.096438 (2012). 22723305

[b6] Jaffer F. A. & Verjans J. W. Molecular imaging of atherosclerosis: clinical state-of-the-art. Heart, doi: 10.1136/heartjnl-2011-301370 (2013). PMC4147037 24365664

[b7] Vasquez K. O., Casavant C. & Peterson J. D. Quantitative whole body biodistribution of fluorescent-labeled agents by non-invasive tomographic imaging. PLoS One 6, e20594, doi: 10.1371/journal.pone.0020594 (2011). 21731618 PMC3120766

[b8] Nahrendorf M. *et al.* Hybrid *in vivo* FMT-CT imaging of protease activity in atherosclerosis with customized nanosensors. Arterioscler Thromb Vasc Biol 29, 1444–1451, doi: 10.1161/atvbaha.109.193086 (2009). 19608968 PMC2746251

[b9] Hall M. A. *et al.* Comparison of mAbs targeting epithelial cell adhesion molecule for the detection of prostate cancer lymph node metastases with multimodal contrast agents: quantitative small-animal PET/CT and NIRF. J Nucl Med 53, 1427–1437, doi: 10.2967/jnumed.112.106302 (2012). 22872743

[b10] Nishi K. *et al.* Oxidized LDL in carotid plaques and plasma associates with plaque instability. Arterioscler Thromb Vasc Biol 22, 1649–1654 (2002). 12377744 10.1161/01.atv.0000033829.14012.18

[b11] Sigala F. *et al.* Oxidized LDL in human carotid plaques is related to symptomatic carotid disease and lesion instability. J Vasc Surg 52, 704–713, doi: 10.1016/j.jvs.2010.03.047 (2010). 20573470

[b12] van Dijk R. A. *et al.* Differential expression of oxidation-specific epitopes and apolipoprotein(a) in progressing and ruptured human coronary and carotid atherosclerotic lesions. J Lipid Res 53, 2773–2790, doi: 10.1194/jlr.P030890 (2012). 22969153 PMC3494262

[b13] Fefer P. *et al.* The role of oxidized phospholipids, lipoprotein (a) and biomarkers of oxidized lipoproteins in chronically occluded coronary arteries in sudden cardiac death and following successful percutaneous revascularization. Cardiovasc Revasc Med 13, 11–19, doi: 10.1016/j.carrev.2011.08.001 (2012). 22079685

[b14] Tsimikas S., Shortal B. P., Witztum J. L. & Palinski W. *In vivo* uptake of radiolabeled MDA2, an oxidation-specific monoclonal antibody, provides an accurate measure of atherosclerotic lesions rich in oxidized LDL and is highly sensitive to their regression. Arterioscler Thromb Vasc Biol 20, 689–697 (2000). 10712392 10.1161/01.atv.20.3.689

[b15] Shaw P. X. *et al.* Human-derived anti-oxidized LDL autoantibody blocks uptake of oxidized LDL by macrophages and localizes to atherosclerotic lesions *in vivo*. Arterioscler Thromb Vasc Biol 21, 1333–1339 (2001). 11498462 10.1161/hq0801.093587

[b16] Torzewski M. *et al.* Reduced *in vivo* aortic uptake of radiolabeled oxidation-specific antibodies reflects changes in plaque composition consistent with plaque stabilization. Arterioscler Thromb Vasc Biol 24, 2307–2312, doi: 10.1161/01.ATV.0000149378.98458.fe (2004). 15528482

[b17] Briley-Saebo K. C. *et al.* Targeted molecular probes for imaging atherosclerotic lesions with magnetic resonance using antibodies that recognize oxidation-specific epitopes. Circulation 117, 3206–3215, doi: 10.1161/circulationaha.107.757120 (2008). 18541740 PMC4492476

[b18] Briley-Saebo K. C., Cho Y. S. & Tsimikas S. Imaging of Oxidation-Specific Epitopes in Atherosclerosis and Macrophage-Rich Vulnerable Plaques. Curr Cardiovasc Imaging Rep 4, 4–16, doi: 10.1007/s12410-010-9060-6 (2011). 21297859 PMC3018294

[b19] Briley-Saebo K. C. *et al.* *In vivo* detection of oxidation-specific epitopes in atherosclerotic lesions using biocompatible manganese molecular magnetic imaging probes. J Am Coll Cardiol 59, 616–626, doi: 10.1016/j.jacc.2011.10.881 (2012). 22300697 PMC3333483

[b20] Lu T. *et al.* Near-infrared fluorescence imaging of murine atherosclerosis using an oxidized low density lipoprotein-targeted fluorochrome. Int J Cardiovasc Imaging 30, 221–231, doi: 10.1007/s10554-013-0320-9 (2014). 24170262

[b21] Chang S. H. *et al.* Model IgG monoclonal autoantibody-anti-idiotype pair for dissecting the humoral immune response to oxidized low density lipoprotein. Hybridoma 31, 87–98 (2012). 22509912 10.1089/hyb.2011.0058 PMC3326269

[b22] Lin S. A. *et al.* Quantitative Longitudinal Imaging of Vascular Inflammation and Treatment by Ezetimibe in apoE Mice by FMT Using New Optical Imaging Biomarkers of Cathepsin Activity and alpha(v)beta(3) Integrin. Int J Mol Imaging 2012, 189254, doi: 10.1155/2012/189254 (2012). 23119157 PMC3483711

[b23] Waschkau B., Faust A., Schafers M. & Bremer C. Performance of a new fluorescence-labeled MMP inhibitor to image tumor MMP activity *in vivo* in comparison to an MMP-activatable probe. Contrast Media Mol Imaging 8, 1–11, doi: 10.1002/cmmi.1486 (2013). 23109387

[b24] Wallis de Vries B. M. *et al.* Images in cardiovascular medicine. Multispectral near-infrared fluorescence molecular imaging of matrix metalloproteinases in a human carotid plaque using a matrix-degrading metalloproteinase-sensitive activatable fluorescent probe. Circulation 119, e534–536, doi: 10.1161/circulationaha.108.821389 (2009). 19470893

[b25] Jaffer F. A. *et al.* Two-dimensional intravascular near-infrared fluorescence molecular imaging of inflammation in atherosclerosis and stent-induced vascular injury. J Am Coll Cardiol 57, 2516–2526, doi: 10.1016/j.jacc.2011.02.036 (2011). 21679853 PMC3123768

[b26] Calfon M. A. *et al.* *In vivo* Near Infrared Fluorescence (NIRF) Intravascular Molecular Imaging of Inflammatory Plaque, a Multimodal Approach to Imaging of Atherosclerosis. J Vis Exp, doi: 10.3791/2257 (2011). PMC3211114 21847078

[b27] Olafsen T. & Wu A. M. Antibody vectors for imaging. Semin Nucl Med 40, 167–181, doi: 10.1053/j.semnuclmed.2009.12.005 (2010). 20350626 PMC2853948

[b28] Stone P. H., Coskun A. U. & Prati F. Ongoing Methodological Approaches to Improve the *in Vivo* Assessment of Local Coronary Blood Flow and Endothelial Shear Stress: The Devil is in the Details. J Am Coll Cardiol 66, 136–138, doi: 10.1016/j.jacc.2015.05.010 (2015). 26160629

[b29] Verhoeven B. A. *et al.* Athero-express: differential atherosclerotic plaque expression of mRNA and protein in relation to cardiovascular events and patient characteristics. Rationale and design. Eur J Epidemiol 19, 1127–1133 (2004). 15678794 10.1007/s10564-004-2304-6

[b30] Bhatia V. K. *et al.* Complement C1q reduces early atherosclerosis in low-density lipoprotein receptor-deficient mice. Am J Pathol 170, 416–426, doi: 10.2353/ajpath.2007.060406 (2007). 17200212 PMC1762701

[b31] Yoo H. *et al.* Intra-arterial catheter for simultaneous microstructural and molecular imaging *in vivo*. Nat Med 17, 1680–1684, doi: 10.1038/nm.2555 (2011). 22057345 PMC3233646

[b32] Verhoeven B. A. *et al.* Athero-express: differential atherosclerotic plaque expression of mRNA and protein in relation to cardiovascular events and patient characteristics. Rationale and design. European journal of epidemiology 19, 1127–1133 (2004). 15678794 10.1007/s10564-004-2304-6

[b33] Lewis M. J. *et al.* Immunoglobulin M is required for protection against atherosclerosis in low-density lipoprotein receptor-deficient mice. Circulation 120, 417–426, doi: 10.1161/circulationaha.109.868158 (2009). 19620499 PMC2761224

[b34] Vinegoni C. *et al.* Indocyanine green enables near-infrared fluorescence imaging of lipid-rich, inflamed atherosclerotic plaques. Sci Transl Med 3, 84ra45, doi: 10.1126/scitranslmed.3001577 (2011). PMC3112179 21613624

