# Supplementary material for: Near Infrared Fluorescence (NIRF) Molecular Imaging of Oxidized LDL with an Autoantibody in Experimental Atherosclerosis
Source: Sci Rep. 2016 Feb 25;6:21785. doi: 10.1038/srep21785 (PMC4766560; doi:10.1038/srep21785)
Supplement: Supplementary Information [file srep21785-s1.pdf]

NEAR INFRARED FLUORESCENCE (NIRF) MOLECULAR IMAGING OF OXIDIZED LDL WITH AN AUTOANTIBODY IN EXPERIMENTAL ATHEROSCLEROSIS

SUPPLEMENTARY INFORMATION

Authors:

Ramzi Y Khamis MB ChB PhD MRCP [1], Kevin J. Woollard PhD [2], Gareth D. Hyde PhD [1], Joseph J Boyle PhD FRCPath [1], Colin Bicknell MD FRCS [3], Shang-Hung Chang[1] MD PhD, Talat H Malik PhD[2], Tetsuya Hara MD PhD [4], Adam Mauskapf BS [4], David W Granger PhD [5], Jason L. Johnson PhD [6], Vasilis Ntziachristos PhD [4], Paul M Matthews MD DPhil FRCP [7], Farouc A Jaffer MD PhD[4], Dorian O Haskard DM FRCP [1]

Affiliations:

[1] Vascular Sciences Section, National Heart and Lung Institute, Imperial College London

[2] Department of Medicine, Imperial College London

[3] Department of Surgery & Cancer, Imperial College London

[4] Cardiovascular Research Center and Cardiology Division, Massachusetts General Hospital, Harvard Medical School

[5] Biopharm R&D, GlaxoSmithKline, Stevenage, United Kingdom

[6] School of Clinical Sciences, University of Bristol, United Kingdom

[7] Brain Sciences, Department of Medicine, Imperial College London

[#] Corresponding author: Professor Dorian O. Haskard, Vascular Sciences Section, National Heart and Lung Institute, Imperial College, Hammersmith Hospital, London UK; e-mail d.haskard@imperial.ac.uk; Tel: +44 207 594 2719; Fax: +44 207 594 3654

Supplementary Figure 1

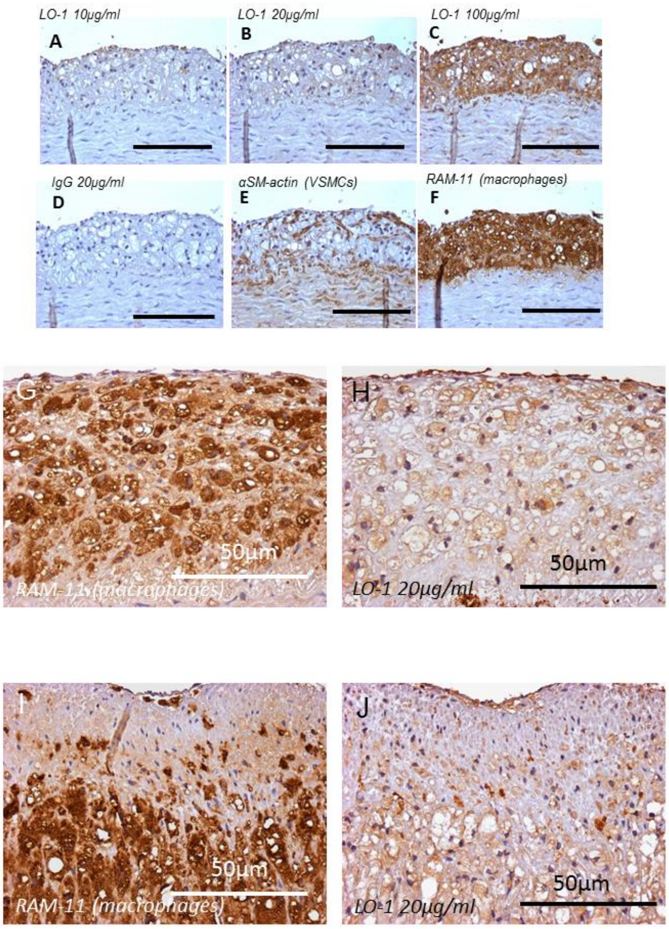

Supplementary Figure 1. ***Immunohistochemical staining of rabbit atherosclerosis with LO1.*** Atherosclerotic sections from the aortic arch of a high fat fed female New Zealand White Rabbit were stained for LO1 reactivity; (**A**-**C**) LO1, but not its isotype control (**D**) bound tissue rich in macrophages, as shown by RAM-11 staining. There was also some staining of free deposits within the plaque, but no clear localization to vascular smooth muscle cells (VSMCs), as identified by anti-smooth muscle actin (aSM-actin, **E**). Scale bars in (**A)** to (**E)** represent 100µm. The same pattern was seen when staining either advanced lesions **(G and H)** with LO1 staining macrophage-rich areas in a thin cap fibro atheroma necrotic core area and deeper in a moderate lesion with a thick cap fibro atheroma **(I and J)**.

Supplementary Figure 2

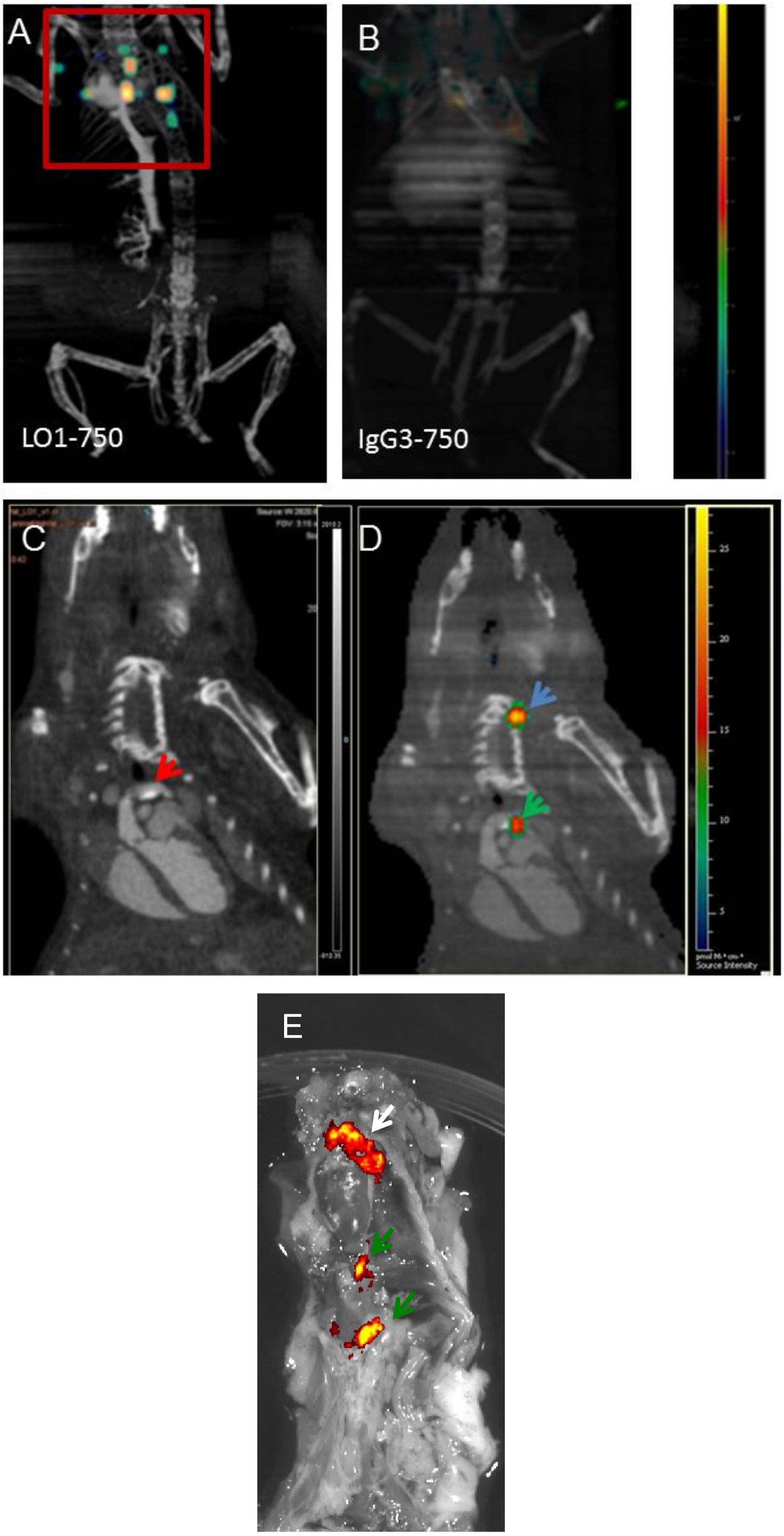

Supplementary Figure 2. ***In vivo*** **aortic imaging of LO1-750 in** ***Ldlr***$^{-/-}$ **mouse model using hybrid IVIS Spectrum/ CT. (A)** LO1-750 localizing to the ascending aorta and aortic arch when scanning the thoracic ROI (red) of a one year old *Ldlr* $^{-/-}$ mouse fed a HF diet for 42 weeks; **(B)** litter mate control showing very little uptake in the same areas following *iv* injection of equivalently-labeled isotype control antibody (IgG3-750); **(C,D)** demonstrates another example, with LO1-750 adjacent to calcium seen on CT at the aortic arch (red arrow) seen on CT **(C)**, co-localizing with LO1-750 signal (green arrow) in (**D**). LO1-750 also identified another hotspot (blue arrow), which was demonstrated to be in the left subclavian artery upon cross-sectional imaging**. (E)** Macroscopic *ex vivo* NIRF of the aorta with its surrounding fatty tissue demonstrates the LO1-750 signal is absent in the fat surrounding the aorta with hot spots in the aortic arch (white arrow), and abdominal aorta (green arrows).

Supplementary Figure 3

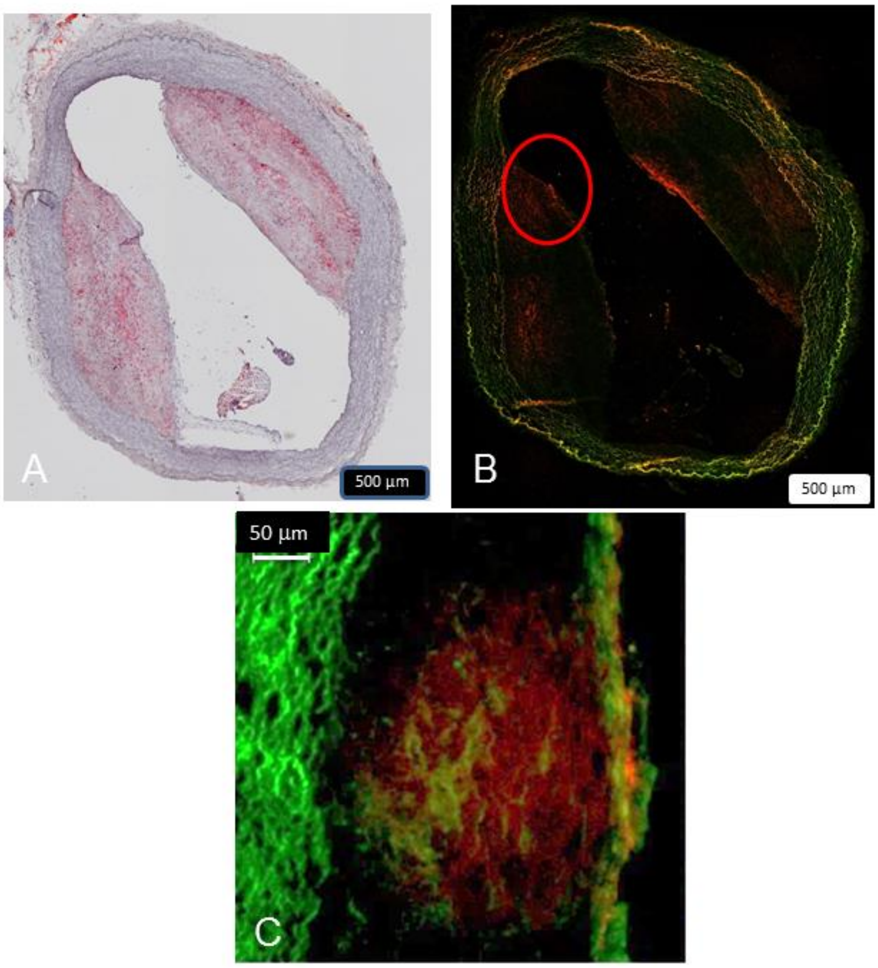

Supplementary Figure 3. ***ex vivo*** **localization of LO1-750 to atherosclerotic plaque in the balloon injured high fat fed rabbit model. (A)** Oil Red O staining of rabbit atherosclerotic plaque demonstrating the lesional lipid content.; **(B)** LO1-750 (Red) localizing to atherosclerotic lesional areas with partial localization to Oil Red O Autofluorescence is in the green FITC channel **(C)** demonstrates higher magnification fluorescence microscopy at the area demarcated by the red oval in B with the LO1-750 signal (red) seen clearly distinct from autofluorescence (green) and in this lesion present in the lesional core extending to the fibrous cap.

Supplementary Figure 4

A QVQLQQSGAELVRPGTSVKVSCKASGYAFTNYLIEWVKQRPGQGLEWIGVINPGSGGT
NYNEKFKGKATLTADKSSSTAYMQLSSLTSEDSAVYFCARSFKWKFDYWGQGTTLTVSS
ASTKGPSVFPLAPSSKSTSGGTAALGCLVKDYFPEPVTVSWNSGALTSGVHTFPAVLQSS
GLYSLSSVVTVPSSSLGTQTYICNVNHKPSNTKVDKKVEPKSCDKTHTC

B

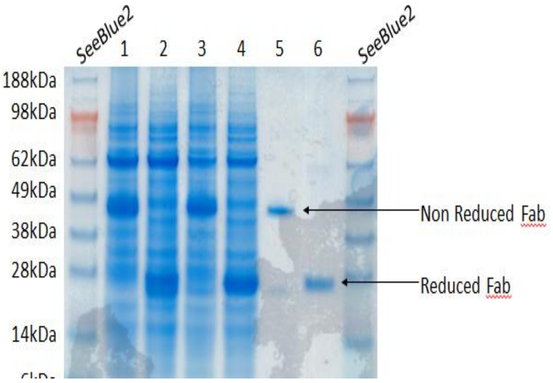

Lane 1- Unpurified LO1-Fab, conc. Non-reduced
Lane 2- Unpurified LO1-Fab conc. Reduced.
Lane 3- Unpurified LO1-Cys_fab. Conc. Non-reduced
Lane 4- Unpurified LO1-Cys-Fab. Conc. Reduced.

C

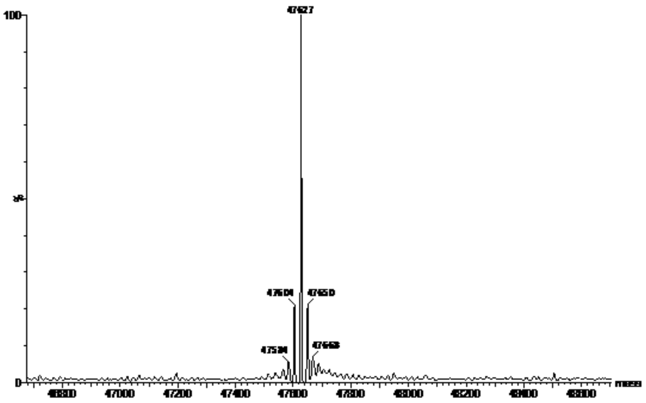

D

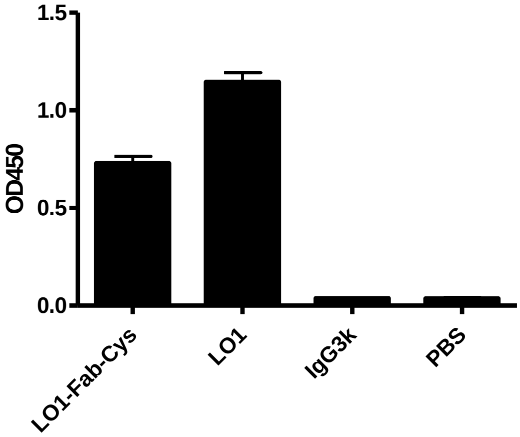

E

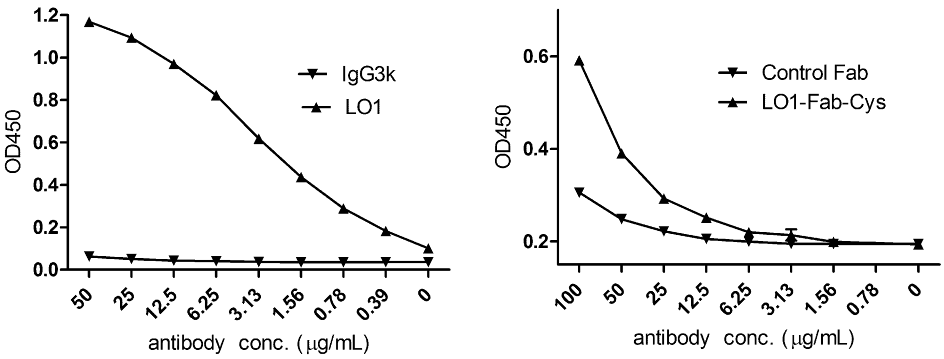

Supplementary Figure 4. Generation of LO1-Fab-Cys. (**A**) shows the amino acid sequence of the heavy chain of molecularly expressed LO1 chimeric Fab with a cysteine tag (Blue- LO1VH; Black- human CH1; Red- Cysteine tag); **(B)** shows the gel electrophoresis of LO1-Cys-Fab affinity purified over an anti- human CH1 column, with protein identified by Simply Blue staining. Lanes 1-4 are unpurified preparations as indicated in the figure. Lane five is a non-reduced purified LO1-Fab-Cys, whilst lane six shows the reduced LO1-Fab-Cys; (**C)** demonstrates the mass spectrum of LO1-Fab-Cys, confirming the mass to be 118Da greater than the theoretical Fab mass (47627 Da vs. 47509 Da), which is consistent with the cysteinylation of the molecule. (**D**) Demonstrates the function of LO1-Fab-Cys in comparison to LO1 and negative controls (on solid phase ELISA), by their ability to capture biotinylated anti-LO1 idiotype H3. **(E)** Titration plots of LO1 (left panel) and LO1-Fab-Cys (right panel) against MDA-LDL, demonstrating the relationship of antibody concentration to binding.

Supplementary movies S1-S4

Supplementary Movie 1. 3D Imaging of LO1-750 uptake in *Ldlr*$^{-/-}$ mice. LO1-750 was injected *iv* into an *Ldlr*$^{-/-}$ mouse that had been fed a high fat diet for 42 weeks, and imaging performed 4 hr later. 3D rotational reconstruction of fused LO1-750 FMT signal with micro-CT. Scale bar represents concentration of LO1-750 in nM.

Supplementary Movie 2.Crosss Sectional Imaging of LO1-750 uptake in *Ldlr-/-* mice. LO1-750 was injected *iv* into an *Ldl*$^{-/-}$ mouse that had been fed a high fat diet for 42 weeks, and imaging was performed 4 hr later. Panels represent coronal, transverse and sagittal dynamic views (left to right) of fused LO1-750 FMT signal with micro-CT. (Same scale as Supplementary Movie 1).

Supplementary Movie 3. Subendothelial localization of LO1-750 following iv injection. An *Ldlr-/-* mouse was injected iv with LO1-750 and phycoerythrin -conjugated anti-CD31, and 4 hr later the aorta was removed for *ex vivo* analysis by confocal microscopy. The movie shows that LO1-750 (red) localized beneath endothelium, as detected by anti-CD31 (green).

Supplementary Movie 4. LO1-Fab-Cys localization in mouse aorta An *Ldlr-/-* mouse was injected iv with LO1-Fab-Cys-750 (50µg), and 4 hr later the aorta was removed for *ex vivo* analysis by confocal microscopy. The movie shows that LO1-Fab-Cys-750 (red) localized beneath subluminal matrix (autofluorescence; Em 520-590, blue)

Supplementary Materials and Methods section:

LO1 and control IgG3

IgG3κ control antibody (I5654, Sigma-Aldrich, UK) was shown by ELISA not to react with LDL or MDA-LDL. Antibodies were buffer-exchanged into PBS with Zeba Desalt Spin Columns (Pierce, Rockfor, IL), and then concentrated to 2 mg/ml prior to labeling, using 100,000 NMWL Centricon centrifugal filter devices (Millipore, Watford, UK). Integrity of the antibodies was monitored using SGS-PAGE followed by Colloidal Blue (Invitrogen, Paisley, UK) staining. An ELISA was used to confirm functional reactivity of LO1 before and after labeling, using MDA-LDL or anti-LO1 idiotype H3 on the solid phase, as previously[1].

Details of the chimeric LO1-Fab-Cys construct

We molecularly expressed the chimeric Fab construct of LO1 with a cysteine-tagged heavy chain (LO1-Fab-Cys). Constructs consisting of human CH1 and CL regions fused respectively to LO1 VH and VL DNA sequences were each cloned into a mammalian expression vector backbone in frame with a DNA sequence encoding an N-terminal secretory signal peptide. The vectors were transfected into HEK293/6E cells and maintained in suspension to express secreted LO1-Fab-Cys molecules. Supernatants were harvested and clarified after 7 days by centrifugation and passing through a 0.22 µm filter. The samples were then concentrated 10-fold using tangential flow prior to purification. Purification was undertaken using an anti-human CH1-IgG purification column (BAC AV, Germany). The purity of LO1-Fab-Cys was confirmed with gel electrophoresis and colloidal blue staining under reducing and non-reducing conditions. Mass spectrometry was used to confirm the mass of the purified LO1-Fab-Cys. Retention of antigen-binding function was confirmed by an ELISA testing the ability of immobilized LO1-Fab-Cys to capture biotinylated anti-LO1

idiotype H3[1]. Inactivation of LO1-Fab-Cys to obtain a negative control was achieved by blocking critical amines required for antigen binding. To achieve this, LO1-Fab-Cys was labeled with an amine reactive dye (Vivo Tag750) to an equivalent degree of labeling (DOL) as with the malemide reactive dye. Deactivation was tested by ELISA as before.

Fluorescence labeling

Labeling of antibodies with the NIRF fluorochrome VivoTag-S 750-MAL (PerkinElmer, Massachusetts USA) was undertaken by adding 20 molar excess of dye to protein, and rotating at 20°C for 2 hr in the dark. Following further purification, and removal of unbound fluorochrome in Zeba Spin Columns, antibodies were tested for both protein and dye content by measuring absorbance at A280 and A750 respectively (Nanodrop 1000, Thermo, Wilmington, DE). The protein concentration in the eluates and the DOL were then established by accounting for both absorbances.

Confocal microscopy on mouse tissues

Tissues were stored at -80°C before use and cryosectioned as previously described [2]. *En face* preparations of aortae were prepared by harvesting the aortae, and cutting them longitudinally. They were then thoroughly cleaned, fixed in 2% formalin, premeabilized by incubating in 0.5% triton to five min, washed and mounted *en face* with the lumen facing up. When staining with antibodies labeled with VivoTag-S 750-MAL, tissues were blocked with 2% bovine serum albumin (BSA), washed and incubated with antibodies (10µg/ml) for 1 hr at 4°C in a humidified chamber. This was followed by a wash using PBS and finalized by nuclear staining with SYTO-24. The slides were then washed and glass cover slips mounted using Fluoromount G. We optimized a Leica SP5 MP inverted confocal microscope for NIRF

imaging at 750 nm. The 633nm laser power was set to 93% or above, and a narrow range (750-800nm) was selected for the emission level to eliminate autofluorescence signal. Most images were obtained using a 10x microscope lens with the frame average of 4.

Confocal microscopy on human tissue

Human carotid endarterectomy specimens were freshly collected with consent and Institutional and National Ethical approval. Specimens were transported and dissected on ice, with a transport time of under 30 min. The tissue was inspected and areas of classical morphology with fibrous cap, lipid core and shoulder identified and snap-frozen in liquid nitrogen and stored at -80°C. The sections were then cryosectioned at -20° C, embedded in OCT and fixed in isopropanol. Staining was as above for mouse tissue, except that macrophages were co-localized with anti-human CD68-FITC (green) (clone KP-1, F7135, Dako, Ely, UK).

Furthermore, well-characterized advanced and intermediate lesions from human coronary sections were also studied. As specified in the AtheroExpress protocol[3], histological sections were classified according to overall appearance into: "atheromatous lesions" containing a large lipid core (>40% of plaque area), high macrophage infiltration with low smooth muscle cell and collagen content, "fibrous lesions" with a small (<40%) or absent lipid core, low macrophage content and high smooth muscle cell and collagen content, and "fibrous-atheromatous lesions" as an intermediate between the two other phenotypes.

Ex vivo confocal analysis of antibody targeting in mice

Following intravenous injection of labeled antibodies, animals were euthanized and the aortae collected and processed as above. The relation of injected antibody to macrophages was obtained by treating aortae with 0.5% triton for 1 hr, followed by staining with rat anti-mouse Macrophages/Monocytes (MOMA-2):Alexa Fluor® 647 (Serotec, Oxford, UK). In some

animals, localization of injected LO1 or control IgG3 in relation to endothelium was studied by injecting phycoerythrin (PE)-conjugated anti-CD31 antibody (Biolegend, San Diego, CA) 10 min prior to humanely killing. To minimize interference between different fluorochromes, each channel was acquired in sequence. To obtain 3D images of full aortae or ROIs, images were acquired as above, with Z stacking and tile scanning. Image analysis for all IHC studies was undertaken using Volocity® 3D Image Analysis Software (PerkinElmer, Massachusetts USA).

IHC in the rabbit model

The aortic arch was removed and fixed in 4% phosphate buffered formaldehyde and then wax-embedded. Briefly, serial 3 μm paraffin sections were dewaxed and rehydrated. Endogenous peroxidase activity was inhibited by incubation with 3% (v/v) hydrogen peroxide. After blocking sections with 20% (v/v) goat serum in PBS, sections were incubated overnight at 4°C with either 20μg/ml LO1, mouse monoclonal antibody against α-smooth muscle actin (Sigma, UK), or mouse monoclonal antibody against rabbit macrophages (RAM11) (Dako, UK), diluted in 1% (w/v) BSA in PBS. Cell nuclei were visualized with haematoxylin. A negative control, where the primary antibody was replaced with mouse IgG at the same dilution, was always included.

Ex vivo fluorescence microscopy on rabbit aortae

Fluorescence microscopy of plaque and normal vessel sections was performed on adjacent sections from fresh frozen rabbit aortae as previously described) [45]. Using an upright epifluorescence microscope (Nikon Eclipse 90i; Tokyo, Japan), fluorescence images were obtained in the NIR channel for FTP11-Cy7 (excitation/emission 710/810 nm; exposure time

50 ms), and FITC channel for autofluorescence (excitation/emission 480/535 nm; exposure time 50 ms [6].

IVIS Spectrum fluorescence imaging and CT (IVIS/CT) in mice

Mice were anesthetized with isoflurane using a vaporizer, and 3D fluorescence images were acquired through Fluorescence Imaging Tomography (FLIT; IVIS® Spectrum, Caliper LifeSciences). We used a GFP filter set (excitation wave length, 710nm ± 15 nm; emission wave length, 820 nm± 15 nm) to detect antibodies conjugated with VivoTag-S 750-MAL. Mice were then transferred anaesthetized in the imaging cassette to the micro-CT imaging suite (Inveon PET-CT, Siemens). CT images were obtained (100µm, 80KVp, 500 µAmp, 120 projections, exposure time 220 ms). Contrast-enhanced high resolution CT localized the aortic root and major arteries in the neck and thorax to guide interpretation. For blood distribution studies, serial tail bleeds at intervals up to 68 hours were performed and samples mixed with equal volumes of heparinized PBS. Blood samples were imaged on a black plate for epifluorescence (ex710, em820) and measured units expressed as total radiant efficiency. Quantification was obtained by non-linear regression curve fit to a known dilution series of the agent. For organ distribution studies, tissues were harvested and imaged in an opaque plate as above. SNR was calculated and expressed as mean ± SEM.

Fluorescence molecular tomography-computed tomography (FMT/CT) in mice

FMT was acquired with an FMT 4000 fluorescence tomography imaging system (PerkinElmer, Massachusetts USA), which is equipped with four lasers and allows acquisition of NIRF from four tracers with distinct excitation and emission wavelengths. Following agent calibration, all animals were injected with a VivoTag-S 750-MAL-conjugated antibody, as well as in selected experiments with MMPSense 645 FAST

(PerkinElmer, MA) (44 nmol/kg) to evaluate matrix metalloproteinase (MMP) activity or AngioSense 680 (PerkinElmer, MA) to allow for identification of intravascular space in isolation of CT. Animals were imaged 4 hr later. In some animals the signals were co-localized with CT images using Siemens Inveon CT scanner (Siemens Healthcare, Erlangen, Germany). An imaging cartridge containing the anesthetized mouse was placed into a custom machined plexiglass holder that supplies isoflurane during imaging. The CT X-ray source with an exposure time of 200 ms was operated at 80 kVp and 500μA. The CT reconstruction was performed with a cone beam reconstruction algorithm, and the pixel values were scaled to Hounsfield units. Contrast-enhanced high resolution CT localized the aortic root and major arteries in the neck and thorax to guide the placement of the ROI in the quantitative fluorescence activity map concomitantly obtained by FMT. Fluorescence and CT image fusion relied on fiducial markers present on the imaging cartridge, and used Amide 1.0.4 (Sourceforge.net).

Two-dimensional NIRF imaging device and catheter for the rabbit model

The two-dimensional NIRF imaging device and catheter apparatus has been fully described previously[5]. Briefly, the intravascular optical probe is capable of performing an over-the-wire pull back intravascular acquisition using a 750 nm laser light and collects the subsequent NIRF emission. The fiber is rotated and translated using mechanical stages to collect fluorescence and generate a 2D NIRF image with longitudinal and angular coordinates.

Intravascular NIRF and IVUS imaging in rabbits

Animals were anesthetized as described previously[5]. A 5-F introducer was inserted into the right carotid artery using fluoroscopic and angiographic guidance. Iodinated contrast was injected and baseline x-ray angiography was recorded using standard cineangiography. An

intravascular ultrasound (IVUS) catheter was inserted over a 0.014-inch guidewire and serial pullbacks were performed (Galaxy IVUS System, Boston Scientific/Scimed, Inc., Natick, Massachusetts) from the iliac bifurcation to the renal arteries, with a pullback length of 100 mm. Next, the monorail NIRF catheter was advanced over the guidewire. To cover the entire IVUS-imaged vessel and understand reproducibility of the NIRF signal profiles, the NIRF catheter was advanced into the iliac artery and 3 to 4 contiguous, 110-mm length pullbacks were performed (rotational speed 30 to 100 rev/min). Animals were then euthanized and the iliacs and aortae were resected. The NIRF catheter was gently reintroduced into lumen of the aorta and *ex vivo* NIRF pullbacks were repeated up to three times. Corresponding images were aligned using iliac bifurcation and renal arteries as landmarks, and radiopaque catheter markers as fiducials. Areas of plaque and normal vessel were confirmed by IVUS images.

Fluorescence reflectance imaging

Resected vessels underwent multiple wavelength FRI (excitation/emission 740/790 nm; Kodak ImageStation 4000, Carestream Health, Inc., Rochester, New York). Exposure times (0.1 to 60 s) generated images that were exported to 16-bit unscaled TIFF files for further analysis with ImageJ version 1.44o (Bethesda, Maryland)[5]. Due to vessel shrinkage after resection, the aorta and the iliac vessels were manually elongated to *in vivo* measured lengths. ROIs were traced manually after visual identification of normal vessel, background, plaque, and injured zones (ImageJ version 1.44o Bethesda, Maryland).

Supplementary references

1 Chang, S. H. *et al.* Model IgG monoclonal autoantibody-anti-idiotype pair for dissecting the humoral immune response to oxidized low density lipoprotein. *Hybridoma* **31**, 87-98 (2012).

2 Lewis, M. J. *et al.* Immunoglobulin M is required for protection against atherosclerosis in low-density lipoprotein receptor-deficient mice. *Circulation* **120**, 417-426, doi:10.1161/circulationaha.109.868158 (2009).

3 Verhoeven, B. A. *et al.* Athero-express: differential atherosclerotic plaque expression of mRNA and protein in relation to cardiovascular events and patient characteristics. Rationale and design. *European journal of epidemiology* **19**, 1127-1133 (2004).

4 Jaffer, F. A. *et al.* Two-dimensional intravascular near-infrared fluorescence molecular imaging of inflammation in atherosclerosis and stent-induced vascular injury. *J Am Coll Cardiol* **57**, 2516-2526, doi:10.1016/j.jacc.2011.02.036 (2011).

5 Hara, T. *et al.* Molecular imaging of fibrin deposition in deep vein thrombosis using fibrin-targeted near-infrared fluorescence. *JACC Cardiovasc Imaging* **5**, 607-615, doi:10.1016/j.jcmg.2012.01.017 (2012).
